# Lutonarin from Barley Seedlings Inhibits the Lipopolysacchride-Stimulated Inflammatory Response of RAW 264.7 Macrophages by Suppressing Nuclear Factor-κB Signaling

**DOI:** 10.3390/molecules26061571

**Published:** 2021-03-12

**Authors:** Ji Yeong Yang, So-Yeun Woo, Mi Ja Lee, Hyun Young Kim, Jin Hwan Lee, Sa-Hyun Kim, Woo Duck Seo

**Affiliations:** 1 Division of Crop Foundation, National Institute of Crop Science (NICS), Rural Development Administration (RDA), Wanju 55365, Korea; yjy90@korea.kr (J.Y.Y.); oxcjswo@gmail.com (S.-Y.W.); esilvia@korea.kr (M.J.L.); hykim84@korea.kr (H.Y.K.); 2 Department of Life Resources Industry, Dong-A University, Busan 49315, Korea; schem72@dau.ac.kr; 3 Department of Clinical Laboratory Science, Semyung University, Jaecheon 27136, Korea

**Keywords:** lutonarin, barley seedlings, NF-κB, anti-inflammatory

## Abstract

Extracts from barley seedlings (BS) have known antioxidant and anti-inflammatory activities. The flavonoid lutonarin (LN) is a component of BS extract and has several known bioactivities. Here, we evaluated LN anti-inflammatory efficacy against lipopolysaccharide (LPS)-stimulated RAW 264.7 macrophages. Lutonarin was isolated from BS by methanol extraction and characterized by ultra-performance liquid chromatography and quadrupole time-of-flight tandem mass spectrometry (UPLC-Q-TOF-MS/MS). Lutonarin did not reduce the viability or enhance the apoptosis rate of RAW 264.7 macrophages at concentrations up to 150 µM. Concentrations within 20–60 µM dose-dependently suppressed the LPS-induced expression, phosphorylation, and nuclear translocation of the inflammatory transcription factor nuclear factor kappa-light-chain-enhancer of activated B cells (NF-κB). Furthermore, LN suppressed the LPS-induced upregulation of proinflammatory cytokines interleukin (IL)-6 and tumor necrosis factor (TNF)-α and of the inflammatory enzyme cyclooxygenase-2 (COX-2) and inducible nitric oxide synthase (iNOS). Lutonarin may be a safe and effective therapeutic agent for alleviation of pathological inflammation.

## 1. Introduction

Inflammation is an essential defense mechanism against various insults, such as wounding, heat, and irradiation, but chronic excessive or chronic inflammation can cause recurrent tissue damage and loss of function as observed in diseases such as rheumatoid arthritis, systemic lupus erythematous, Crohn’s disease, and atopic dermatitis [1]. Furthermore, inflammation plays a central role in tumor development [2].

Nuclear factor kappa-light-chain-enhancer of activated B cells (NF-κB) is a transcription factor that regulates inflammation by activating a host of downstream effectors [3]. The canonical NF-κB activation pathway is regulated by various extracellular stimuli including lipopolysaccharide (LPS) [4], a principal component of the Gram-negative bacteria outer membrane and a potent stimulator of macrophages, the main phagocytic cells of the innate immune system [5]. When LPS stimulates macrophages, the NF-κB negative regulator IκBα is rapidly phosphorylated and degraded, allowing for NF-κB phosphorylation by upstream signaling cascades and translocation to the nucleus [6]. Then, nuclear NF-κB drives the expression of cytokines such as interleukin (IL)-1β, IL-6, and tumor necrosis factor (TNF)-α and of inflammatory enzymes such as cyclooxygenase-2 (COX-2) and inducible nitric oxide synthase (iNOS), factors involved in pathogenesis of inflammatory disease [7].

Flavonoids are a diverse group of naturally occurring secondary metabolites found in plants with demonstrated health benefits against cardiovascular disease and cancer [8]. Additionally, flavonoids have been known to have anti-inflammatory activity [9]. Therefore, flavonoids could be used in dietary or pharmaceutical supplements to prevent and treat pathological inflammation. 

Lutonarin (LN, isoorientin-7-O-glucoside, PubChem CID 44257976) is a plant flavonoid enriched in barely seedlings (BSs) with known antioxidant [10] and anti-neuraminidase [11] activity. Here, we demonstrate that LN also inhibits the inflammatory response of LPS-treated RAW 264.7 macrophages by reducing expression of proinflammatory mediators via blockade of NF-κB signaling.

## 2. Materials and Methods

### 2.1. Isolation and Quantification of Lutonarin (LN) from Barley Seedlings (BS) Methanol Extract

Lutonarin (LN, isoorientin-7-O-glucoside, PubChem CID 44257976) was isolated as previously reported [12] with some modifications. The methanol extracts were purified by multiple-preparation reversed-phase HPLC using 0.1% TFA in water (A) and 0.1% TFA in acetonitrile (B) as mobile phases at a flow rate of 25 mL/min. The gradient elution steps were as follows: 0 min, 0% B; 0–35 min, 0–15% B; 35–80 min, 15–100% B, followed by 25 min recycle time. Elution was monitored at 245, 280, and 325 nm absorbance using a photodiode array (PDA). The purity of LN was determined using the Acquity ultra-performance liquid chromatography photodiode detector and quadrupole time-of-flight mass spectrometry (UPLC-PDA-Q/TOF-MS) system (Waters, Milford, MA, USA) operating in negative ion modes in the following conditions: capillary voltage 2.3 kV and cone voltage 50 V. Nitrogen was used as the desolvation gas, with a desolvation temperature of 350 °C, flow rate of 780 L/h, and source temperature of 150 °C. The capillary and cone voltages were set to 3290 and 55 V, respectively. The Q-TOF premier^TM^ was operated in v mode with a 9000 mass-resolving power. Data for each test sample were collected from 100 to 1500 Da with a 0.25 s scan time and 0.01 s inter-scan delay over 15 min. Leucine-enkephalin was used as the reference compound (*m*/*z* 554.2615 in the negative mode) with an infusion flow rate of 1 μL/min. The classical method for glycol conjugate identification was adopted to designate the fragment ions [13].

The major compounds of barley seedlings (BS) were profiled and quantitated as previously reported [11] by ultra-performance liquid chromatography (UPLC, Waters, Milford, MA, USA). In brief, the temperature of the Waters ACQUITY BEH C18 column (particle size 1.7 µm, 2.1 × 100 mm, Waters, Milford, MA, USA) was maintained at 35 °C and detection was observed with a PDA detector at 335 nm. Mobile phases A and B were 0.1% TFA and acetonitrile, respectively, and the gradient elution steps were as follows: 0–3 min, 3% B; 3–10 min, 3–15% B; 10–13 min, 15–30% B; 13–15 min, 30–50% B; washing with 90% B to 18 min, followed by a 2 min recycle step at a flow rate of 0.5 mL/min. The BS compound peaks were identified relative to retention times of known standards.

### 2.2. Cell Culture 

The macrophage cell line RAW 264.7 was purchased from the Korean Cell Line Bank (KCLB, Seoul, Korea) and cultured in Dulbecco’s modified Eagle’s medium (DMEM, Gibco, Grand Island, NY, USA) supplemented with 10% fetal bovine serum (FBS, Gibco, Grand Island, NY, USA) and 1% penicillin-streptomycin solution (100 U/mL penicillin and 100 µg/mL streptomycin in 0.85% NaCl, Invitrogen, Carlsbad, CA, USA). The cells were incubated in a humidified environment at 37 °C under a 5% CO_2_ atmosphere.

### 2.3. Colorimetric MTT Assay

The (4,5-dimethylthiazol-2-yl)-2,5-diphenyltetrazolium bromide) assay (MTT assay) was used to evaluate the effects of LN on RAW 264.7 cell viability. Briefly, cells were seeded in 24-well plates at 1 × 10^5^ cells/well and incubated for 24 h. Then, the cells were pretreated with different concentrations of LN for 4 h and subsequently stimulated with 1 µg/mL LPS for 24 h. Cells were, then, incubated with MTT working solution for 4 h at 37 °C, and the formazan accumulation from viable cells measured at 475 nm absorbance on a SpectraMax M5 fluorescence spectrophotometer (Molecular Devices, San Jose, CA, USA). Cell viability (%) was estimated according to the equation ((A_sample_/B_blank_) × 100), where A_sample_ and B_blank_ are the absorbances of LPS-treated and vehicle (DMSO)-treated cultures, respectively.

### 2.4. Electrophoretic Mobility Shift Assay (EMSA) 

To assess NF-κB binding to promoter sequences, nuclear extracts from RAW 264.7 macrophages were prepared, according to the method of Staal et al. (1990) and subjected to electrophoretic mobility shift assays (EMSAs) using the biotin end-labeled double-started NF-κB probe (5′-biotin-AGTTGAGGGGACTTTCCCAGGC-3′). Nuclear protein extract (4 mg) was incubated on ice for 1 h with 0.25 pmole ^32^P-end-labelled oligonucleotide in binding buffer containing 20 mM HEPES (pH 7.5), 4% Ficoll, 0.5 mg/mL poly-DIDC, 0.1 mM MgCl_2_, and 0.1 mM DTT. The nuclear extract complexed with NF-κB probe was separated from free oligonucleotides by 4% non-denaturing polyacrylamide gel electrophoresis in TBE buffer (89 mM Tris-HCl, pH 8.0, 89 mM boric acid, and 2 mM EDTA). The biotin–DNA complex was detected using the enhanced LightShift Chemiluminescent EMSA Kit (Panomics, Fremont, CA, USA) and visualized using a CAS-400SM Davinch-Chemi chemiluminescence imaging system (Seoul, Korea).

### 2.5. NF-κB Vector Transfection and Luciferase Assays

RAW 264.7 macrophage cells were transfected with a NF-κB luciferase promoter-reporter construct (pGL4.32 [luc2P/NF-kB-RE/Hygro]) in serum-free DMEM using FuGENE 6 (Promega, Madison, WI, USA) as a transfection reagent, according to the manufacturer’s instructions. Transfected cells were resuspended in DMEM containing 10% FBS and allowed to recover for 5 h. Then, the cells were switched to DMEM containing 1% penicillin-streptomycin for 24 h prior to experiments. Cells were pretreated with LN for 4 h, and then stimulated with 1 µg/mL LPS for 24 h. Total protein was isolated using passive lysis buffer (Promega, Madison, WI, USA) and luciferase activity determined by a microplate reader (Molecular Devices, San Jose, CA, USA). Relative luciferase activity was reported as fold changed as compared with untreated controls normalized for transfection efficiency.

### 2.6. Protein Extraction and Western Blotting Assay

Cells were collected and lysed in RIPA buffer containing 20 mM Tris-HCl, pH 7.5, 150 mM NaCl, 1 mM EDTA, 1 mM EGTA, 1% NP-40, 1% sodium deoxycholate, 2.5 mM sodium pyrophosphate, 1 mM β-glycerophosphate, 1 mM Na_3_VO_4_, and 1 µg/mL leupeptin (Cell Signaling Technology, Beverly, MA, USA). Then, the lysates were centrifuged at 12,000× *g* for 15 min at 4 °C. Supernatants were collected and total protein concentrations were determined using the DC protein assay (Bio-Rad, Hercules, CA, USA), according to the manufacturer’s instructions. Samples were separated by 10–15% SDS–polyacrylamide gel electrophoresis and transferred to nitrocellulose membranes (Millipore, Burlington, MA, USA) using a semidry electroblotting system (Bio-Rad, Hercules, CA, USA). Nitrocellulose membranes were incubated overnight at 4 °C with primary antibodies against the following proteins: COX-2, iNOS, NF-κB p65, phosphorylated NF-κB p65 (p-p65), IκBα, and p-IκBα. After washing off the primary antibody, the blots were incubated for 2 h with mouse or rabbit secondary antibodies (Cell Signaling Technology, Beverly, MA, USA). Immunoreactive protein bands were detected using an enhanced chemiluminescence (ECL) detection kit and captured with the CAS-400SM Davinch-Chemi chemiluminescence imaging system. 

### 2.7. RNA Isolation and Reverse Transcriptase Polymerase ChainRreaction (RT-PCR) Assay

Total RNA was isolated using Trizol reagent (Invitrogen, Carlsbad, CA, USA), according to the method for mammalian cell culture, and TNF-α, IL-6, and IL-1β mRNA levels estimated by reverse transcriptase polymerase chain reaction (RT-PCR). Briefly, 1 µg RNA was reverse transcribed using the QIAGEN one-step RT-PCR kit (QIAGEN, Hilden, Germany) and the following primers: actin (forward primer 5′-GTGGGCCGCCCTAGGCACCAG-3′ and reverse primer 5′-GGAGGAAGAGGATGCGGCAGT-3′), TNF-α (forward primer 5′-TTGACCTCAGCGCTGAGTTG-3′ and reverse primer 5′-CCTGTAGCCCACGTCGTAGC-3′), IL-6 (forward primer 5′-GTACTCCAGAAGACCAGAGG-3′ and reverse primer 5′-TGCTGGTGACAACCACGGCC-3′), and IL-1β (forward primer 5′-CAGGATGAGGACATGAGCACC-3′ and reverse primer 5′-CTCTGCAGACTCAAACTCCAC-3′). All RT-PCR runs were conducted using an Applied Biosystems thermocycler (Foster City, CA, USA) with the following settings: 35 cycles of 94 °C for 1 min (denaturing), 50–68 °C for 1 min (annealing), and 72 °C for 1 min (primer extension). The reaction products were subjected to electrophoresis on 1.5% agarose gels and stained with SYBR safe^TM^ (Invitrogen, Carlsbad, CA, USA).

### 2.8. Statistics

All data were analyzed by one factor or two-factor analysis of variance (ANOVA) using SAS software (Abacus Concepts Inc., Berkeley, CA, USA). A value of *p* < 0.05 was considered to be statistically significant for all tests.

## 3. Results

### 3.1. Confirmation of LN Content in BS Methanol Extract

**Figure 1:**
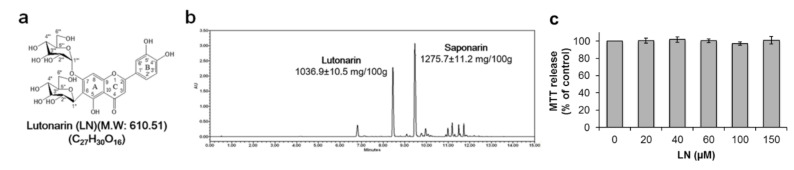
Identification of lutonarin (LN) in methanol extract of barley seedlings. (**a**) Chemical structure of LN; (**b**) Representative ultra-performance liquid chromatography and photodiode array (UPLC-PDA) chromatogram of barley sprout extract showing that saponarin and LN are the major constituents; (**c**) RAW 264.7 macrophages were pretreated with the indicated LN concentrations for 4 h, and then cultivated for 24 h with or without 1 µg/mL lipopolysaccharide (LPS). MTT assay of viable cell number after LN treatment. Values are presented as the mean ± SEM of three independent replicates. Lutonarin did not reduce viable cell number within the tested concentration range.

The major compounds in BS methanol extract, as revealed by ultra-performance liquid chromatography and photodiode array (UPLC-PDA), were saponarin (SN, isovitexin-7-O-glucoside, 1275.7 mg/100 g) and LN (molecular weight (M.W.) = 610.51, 1036.9 mg/100 g) (Figure 1a,b). We previously demonstrated that SN suppresses the inflammatory response of LPS-treated RAW 264.7 cells (Seo, et al., 2014); however, the anti-inflammatory efficacy of LN remains unclear. Therefore, we isolated LN from BS, and then verified its chemical structure by UPLC quadrupole time-of-flight mass spectrometry (QToF/MS) analysis relative to known mass spectra (Figure S1). 

### 3.2. Cytotoxicity of LN against LPS-Stimulated and Unstimulated RAW 264.7 Macrophages

The cytotoxicity of LN against LPS-stimulated and unstimulated RAW 264.7 macrophages was examined by MTT and crystal violet staining assays for cell viability (Figure 1c and Figure S2). Effects on cell viability were negligible in the concentration range of 20 to 150 µM in both the presence and absence of LPS. Furthermore, LN had little effect on apoptosis rate as evaluated by dual PI and Annexin V-FITC staining, and subsequent flow cytometry (Figure S2). Control cells were negative for both PI (which binds to genomic DNA in cells with loss of plasma membrane integrity during late apoptosis) and Annexin V (which binds with high affinity to phosphatidylserine translocated from the inner to the outer membrane leaflet during early apoptosis). Early apoptosis was slightly increased (0.63%) by LPS and decreased dose-dependently by LN pretreatment (0.55% with 40 µM LN and 0.26% with 60 µM LN), although the difference from untreated controls did not reach significance. Thus, LN showed minimal toxicity against LPS-stimulated and unstimulated RAW 264.7 macrophages within the dose range employed for subsequent experiments. 

### 3.3. Inhibitory Effect of LN on LPS-Induced NF-κB Activation

**Figure 2:**
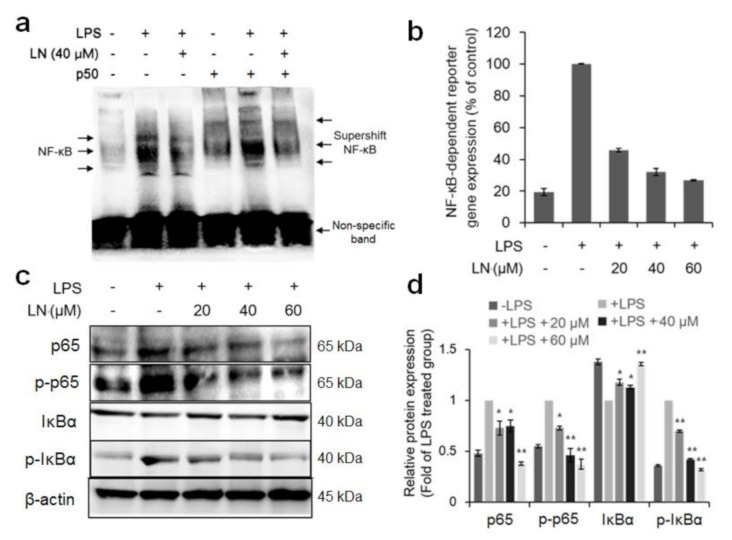
Lutonarin (LN) inhibited LPS-stimulated NF-κB activation in RAW 264.7 macrophages. Cells were pretreated with the indicated LN concentrations for 4 h, then cultivated for 24 h with 1 µg/mL LPS. (**a**) Effect of LN on NF-κB DNA binding. Nuclear extracts of cells treated as indicated (LN, LPS, and/or anti-p50) were prepared and analyzed for NF-κB activation by electrophoretic mobility shift assay (EMSA). LN dose-dependently reduced the supershift in the NF-κB p50 component band; (**b**) Effect of LN on LPS-activated NF-κB-dependent reporter gene expression. LN dose-dependently blocked LPS-induced luciferase activity driven by the NF-κB gene promoter in RAW 264.7 macrophages; (**c**) LN also suppressed the expression of NF-κB subunit p60, the negative regulator IkBa, and corresponding phosphorylated forms as evidenced by Western blot analysis. β-actin was used as the gel-loading control; (**d**) Densitometric analysis of Western blots. LN dose-dependently reversed the LPS-induced elevations in p65, p-p65, and p-IκBα and reversed the LPS-induced reduction in IκBα suggesting inhibition of degradation. Values are presented as the mean ± SEM of three independent replicates (* *p* < 0.05 and ** *p* < 0.01 by ANOVA).

NF-κB activation is a critical regulator of inflammation in response to a variety of extracellular stimuli including microbial factors such as LPS [4]. To investigate whether LPS-induced NF-κB activation is suppressed by pretreatment with LN, the NF-κB binding activity in nuclear extracts from RAW 264.7 cells was analyzed by electrophoretic mobility shift assay (EMSA). Nuclear factor-κB is a protein dimer composed of several distinct subunit isoforms, and the various dimer combinations bind differentially to genomic sequences [14], therefore, multiple DNA–protein bands appear on polyacrylamide gels (Figure 2a). To confirm that the band visualized by EMSA in LPS-treated cells was indeed NF-κB, we incubated the nuclear extracts from LPS-stimulated macrophages with antibodies targeting p50, one of the major components of NF-κB [7]. This major band was shifted to a higher molecular mass (supershift), suggesting that the LPS-activated complex in these cells consists of p50 subunits (Figure 2a). The LPS-treated cells seemed to increase the activation of NF-κB as compared with the untreated controls, as evidenced by subunit expression in the nuclear extract, while pretreatment of 40 µM LN reduced NF-κB expression and the anti-p50 supershift (Figure 2a). Furthermore, LPS increased the luciferase activity in NF-κB luciferase reporter vector-transfected macrophages by approximately five-fold as compared with the untreated cells, while LN pretreatment dose-dependently reduced this activity (Figure 2b). The NF-κB p65 subunit is also activated by LPS in macrophages [7] and Western blot analysis revealed that LN pretreatment dose-dependently attenuated p65 and phosphorylated p65 expression levels (Figure 2c). Additionally, IκBα phosphorylation and ensuing degradation are required for NF-κB activation [15] and we found that LPS-stimulated IκBα degradation and phosphorylation were suppressed by LN pretreatment (Figure 2c). Collectively, these results indicate that LN effectively suppresses LPS-induced NF-κB activation by inhibiting p65 expression and phosphorylation as well as IκBα phosphorylation and degradation.

### 3.4. Inhibitory Effect of LN on LPS-Induced Inflammation

**Figure 3:**
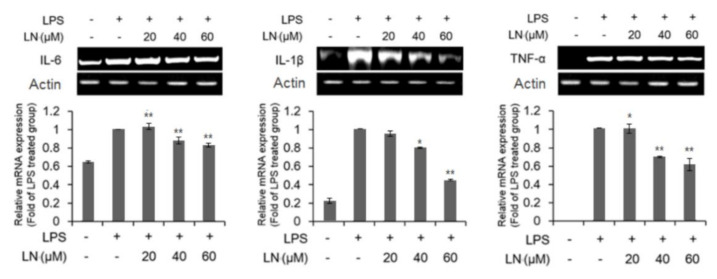
Lutonarin (LN) reversed LPS-induced elevations in proinflammatory cytokines. Macrophages were pretreated with the indicated LN concentration for 4 h, and then cultivated with 1 µg/mL LPS for 24 h. The mRNA levels were measured by reverse transcriptase polymerase chain reaction (RT-PCR). Actin was used as an internal control. Values are expressed as mean ± SEM of three independent replicates and compared to cells treated with LPS only. * *p* < 0.05 and ** *p* < 0.01 by ANOVA.

**Figure 4:**
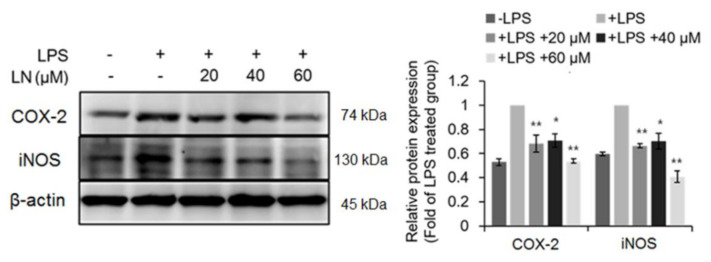
Lutonarin reversed the LPS-induced upregulation of anti-inflammatory enzymes COX-2 and iNOS. RAW 264.7 cells were incubated with the indicated concentration of lutonarin (LN) for 4 h, and then treated with 1 µg/mL LPS for 24 h. Proteins were extracted, separated by SDS-PAGE, and quantified by Western blotting. Representative image of data is shown. A graph illustrates the density of the bands as compared with that of cells treated with LPS-only group. Data were from the three independent experiments and analyzed by ANOVA (* *p* < 0.05 and ** *p* < 0.01).

This inhibition of LPS-induced NF-κB activation suggests that LN may also be able to reduce the expression of downstream proinflammatory cytokines in response to LPS. Indeed, the LPS-induced production levels of IL-6 and TNF-α were dose-dependently reduced by LN pretreatment, while there was no effect on IL-1β expression (Figure 3). Furthermore, Western blot analysis revealed that LN dose-dependently suppressed LPS-induced upregulation of COX-2 and iNOS expression (Figure 4). Thus, LN appears to block the LPS-triggered inflammatory response of RAW 264.7 macrophages by inhibiting activation of the NF-κB signaling pathway. 

## 4. Conclusions

Plant flavonoids have beneficial health effects including anti-inflammatory activity [16] and LN is the main flavonoid constituent of BS (1036.9 mg/100 g, Figure 1). In the current study, we show that LN has no deleterious effects on cell viability at concentrations up to 150 µM (Figure 1b and Figure S2), and that this dose range prevents the LPS-induced inflammatory response in macrophages, as evidenced by suppression of NF-κB signaling and concomitant downregulation of proinflammatory cytokines (IL-6 and TNF-α) and inflammatory enzymes (COX-2 and iNOS). On the basis of this potent anti-inflammatory efficacy, high bioavailability, and non-toxicity, we suggest LN as a potential candidate for clinical trials against chronic inflammatory diseases. 

The transcription factor NF-κB is the main effector of the cellular inflammatory response [17]. Both EMSAs and NF-κB reporter gene expression assays indicated that LPS-induced NF-κB–DNA binding (Figure 2a) and transcriptional activity (Figure 2b) were reduced dose-dependently by LN pretreatment. NF-κB is a heterodimer consisting of different p50 and p65 subunit isoforms [7]. Under basal conditions, the dimer is sequestered in the cytoplasm by IκBα [18]. Phosphorylation and ensuing degradation of IκBα upon LPS stimulation allows for p65 phosphorylation and subsequent translocation to the nucleus, where it binds to the promoters of multiple inflammatory mediators such as cytokines [6]. Lutonarin also inhibited IκBα phosphorylation and prevented degradation (Figure 2c), thereby promoting the retentions of NF-κB in the cytoplasm, suppressed the transcription and phosphorylation (activation) of p65 (Figure 2c), and the expression of nuclear p50 (Figure 2a). This dose-dependent suppression of NF-kB signaling resulted in reduced LPS-induced upregulation of proinflammatory factors IL-6, TNF-α, COX-2, and iNOS (Figure 3 and Figure 4), and blockade of these factors is an effective therapeutic strategy against inflammatory disease [19]. Therefore, further analysis of the mechanism regulating LN-induced inhibition is warranted. 

Our previous study demonstrated that saponarin, the other major component of BS methanol extract, inhibits inflammation via NF-κB suppression [20]. The current results suggest that BS extract or isolated LN may be an effective and safe therapeutic agent against pathological inflammation.

## Data Availability

The data that support the findings of this study are available from the corresponding author upon reasonable request.

## Supplementary Materials

Figure S1: UPLC-Q-ToF-MS/MS mass spectrum of lutonarin (LN), Figure S2: Lutonarin (LN) showed little toxicity against lipopolysaccharide (LPS)-treated and untreated RAW 264.7 macrophages within the working concentration range: (**a**) Crystal violet staining assay. Lutonarin did not reduce viable cell number within the tested concentration range; (**b**) FACS analysis of macrophages treated as described and stained with the apoptosis markers propidium iodide (PI) and FITC-conjugated Annexin V. Note that >99% of cells are P1/Annexin V negative (non-apoptotic) while <1% are in the early or late stages of apoptosis, even at the highest LN dose tested.
