# Supplementary material for: Lutonarin from Barley Seedlings Inhibits the Lipopolysacchride-Stimulated Inflammatory Response of RAW 264.7 Macrophages by Suppressing Nuclear Factor-κB Signaling
Source: Molecules. 2021 Mar 12;26(6):1571. doi: 10.3390/molecules26061571 (PMC7999162; doi:10.3390/molecules26061571)
Supplement: Supplementary file 1 [file molecules-26-01571-s001.pdf]

Supplementary Materials

Annexin V/PI Staining

The effect of LN on macrophage apoptosis rate was examined using the FITC Annexin V Apoptosis Detection kit (Becton Dickinson, Franklin Lakes, NJ, USA), according to the manufacturer's instructions. Briefly, 1 × 10^6 cells were harvested, washed with phosphate-buffered saline (PBS), and resuspended in 500 µL binding buffer. Next, 5 µL Annexin V-FITC and 1 µL PI from the kit were added to the cell suspension. Flow cytometric analysis was performed immediately after staining using a FACS Calibur system (Becton Dickinson, Franklin Lakes, NJ, USA) and CellQuest software (Becton Dickinson, Franklin Lakes, NJ, USA). Cells negative for both Annexin V and PI were defined as none apoptotic, those positive for Annexin V as in the early stage of apoptosis, and those positive for both Annexin V and PI in the late stage of apoptosis.

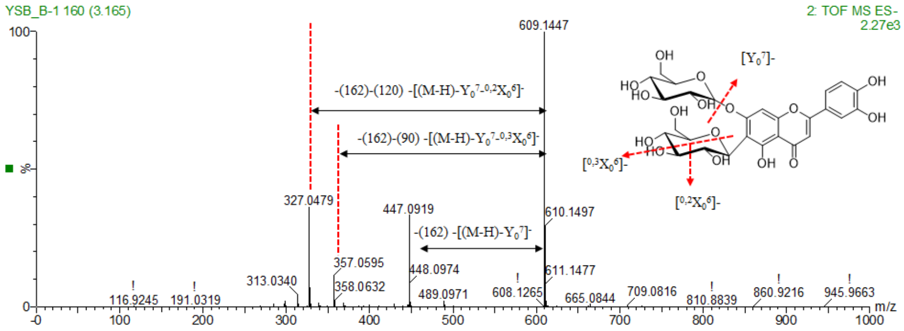

Figure S1. UPLC-Q-ToF-MS/MS mass spectrum of lutonarin (LN).

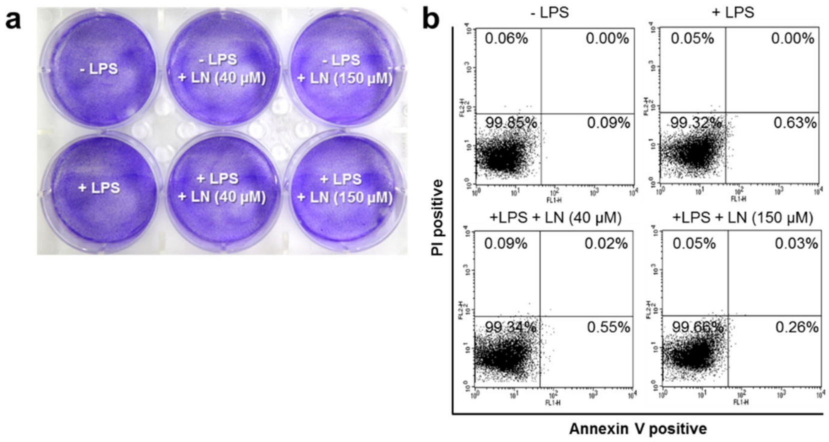

Figure S2. Lutonarin (LN) showed little toxicity against lipopolysaccharide (LPS)-treated and untreated RAW 264.7 macrophages within the working concentration range. (**a**) Crystal violet staining assay. Lutonarin did not reduce viable cell number within the tested concentration range; (**b**) FACS analysis of macrophages treated as described and stained with the apoptosis markers propidium iodide (PI) and FITC-conjugated Annexin V. Note that >99% of cells are P1/Annexin V negative (non-apoptotic) while <1% are in the early or late stages of apoptosis, even at the highest LN dose tested.
